# Locomotor adaptations to prolonged step-by-step frontal plane trunk perturbations in young adults

**DOI:** 10.1371/journal.pone.0203776

**Published:** 2018-09-20

**Authors:** Eric R. Walker, Allison S. Hyngstrom, Tanya Onushko, Brian D. Schmit

**Affiliations:** 1 Department of Biomedical Engineering, Marquette University and the Medical College of Wisconsin, Milwaukee, WI, United States of America; 2 Department of Physical Therapy, Marquette University, Milwaukee, WI, United States of America; University of Illinois at Urbana-Champaign, UNITED STATES

## Abstract

The purpose of this study was to quantify the magnitude and time course of dynamic balance control adaptations to prolonged step-by-step frontal plane forces applied to the trunk during walking. Healthy young participants (n = 10, 5 female) walked on an instrumented split-belt treadmill while an external cable-driven device applied frontal plane forces to the trunk. Two types of forces were applied: 1) forces which accentuated COM movement in the frontal plane (destabilizing) and 2) forces which resisted COM movement in the frontal plane (stabilizing). We quantified dynamic balance control using frontal plane measures of (1) the extent of center of mass (COM) movement over a gait cycle (COM sway), (2) the magnitude of base of support (step width), and (3) cadence. During destabilizing force conditions, COM sway, step width, and cadence increased. In response to stabilizing force conditions, COM sway decreased. In addition, during destabilizing balance conditions participants made quicker adaptations to their step width compared to the time to adapt to stabilizing forces. Taken together, these results provide important insight into differences in dynamic balance control strategies in response to stabilizing and destabilizing force fields.

## Introduction

Adaptations to dynamic balance control are an important component of adjusting to novel walking environments. Generally, maintaining balance during walking is a challenging control task for the central nervous system due to the bipedal nature of human locomotion. Balance is achieved by maintaining the body’s center of mass (COM) within the base of support; however, the height of the body’s COM from the ground and the constantly changing base of support complicate this task [1]. Previous modeling and human experimentation has demonstrated that human locomotion is passively stable in the sagittal plane, suggesting active balance control primarily focuses on the unstable fontal plane [2, 3]. Perturbations of visual feedback [4] and oscillation of the support surface [5] during treadmill walking support this theory. These studies show that neurologically intact individuals have a greater volitional response to discrete perturbations in the frontal plane as opposed to sagittal plane to maintain balance. However, little is known regarding dynamic balance responses to prolonged step-by-step perturbations in the frontal plane which are also functionally relevant. The goal of this study was to apply prolonged step-by-step frontal plane forces to the trunk to identify how individuals adjust their walking cycle to maintain dynamic balance.

Successful movement control requires step-by-step adjustments of the motor plan to meet the specific demands of the task and the environment. These adjustments usually occur very rapidly for walking, making it difficult to gain insight into the underlying motor control strategy. However, performance of skilled movements within a novel environment can provide insight into how these movements are controlled. For example, stepping on a rotating support surface produces a podokinetic after-rotation, in which individuals when blindfolded, produce a curved over ground walking trajectory when the support surface rotation stops [6]. Further analysis of these aftereffects have provided additional insight into the role of the vestibular system in locomotor control [7], as well as support for a single neural center responsible for locomotor trajectory control [8]. Split-belt treadmill adaptation studies, where each leg is moving at a different speed, have provided further insight into locomotor control. Different adaptation rates for intralimb and interlimb locomotor parameters to split-belt walking suggest that separate neural networks are responsible for the control of these parameters during walking [9]. Additionally, altering the level of attention to the adaptation task affects adaptation rates of spatial and not temporal parameters, suggesting spatial parameters may be controlled by cortical centers during walking [10]. Finally, studies using a computer assisted rehabilitation environment, which provide frontal and sagittal plane translations of a walking surface and visual feedback, have demonstrated steady state gait adaptations in individuals with and without neurological injury [11–14]. Perturbations from these studies did not specifically consider dynamics of trunk motion, which can greatly impact control of COM. In the current study, we posited that altering the dynamics of trunk motion during walking would provide a mechanism to evaluate adaptations to dynamic balance control.

Medial-lateral forces applied to the trunk have been used to create a novel balance environment to study adaptations to dynamic balance control in the frontal plane. Lateral trunk perturbations have been previously used to characterize the utilization of a lateral foot placement strategy to maintain balance during walking [15]. However, the perturbations used by Hoff et al. [15] were of a short duration and only the initial corrective response to the perturbation was characterized.

In this study, we created a novel dynamic balance environment using step-by-step, cyclical forces applied to the trunk while study participants stepped on an instrumented treadmill. The effects of stabilizing and destabilizing forces were tested to identify adaptations in both directions. We hypothesized that individuals would increase step width in response to destabilizing forces, and would decrease step width for stabilizing forces. Furthermore, we anticipated that adaptations to destabilizing forces would occur rapidly to prevent a loss of balance.

## Materials and methods

Ten individuals (5 males, 5 females, ages 21–30) with no reported neurological injury or disease participated in this study. The Marquette University Institutional Review Board approved all experimental procedures, and written informed consent was obtained from all individuals prior to participating in this study.

Fifteen passive infrared reflective markers were placed at anatomical locations according to the Conventional Gait Model[16] (Plug-In-Gait in Vicon) to capture lower extremity movement. Additionally, markers were placed bilaterally on the wrist, elbow, shoulder, front and back of the head, and also on the C7 vertebra to quantify movements of the upper extremity and trunk. Marker locations were recorded at 100 Hz using an 8-camera Vicon motion capture system (Vicon Motion Systems Ltd, Oxford, UK). Ground reaction forces were recorded from an instrumented, split-belt treadmill (FIT, Bertec, Colombus, OH). Note that the belts moved at the same speed, but independent measurements from each belt were used for gait kinetics. Ground reaction forces and moments (six degrees of freedom) were measured independently for each belt. A custom handle, instrumented with a six degree of freedom load cell (AMTI, MC3A-250, Watertown, MA), was attached to a front handrail of the treadmill to quantify handrail hold forces. Handle forces were amplified at 1,000 V/V, and low-pass filtered at 500 Hz prior to collection (Gen5, AMTI Inc., Watertown, MA). Perturbation forces were measured using a load cell (MLP-300, Transducer Techniques Inc., Temecula, CA) attached in line with the cable. Signals were amplified at 450V/V and low-pass filtered at 250 Hz prior to collection (TMO-1-24, Transducer Techniques Inc., Temecula, CA). Ground reaction forces, handle forces, and cable perturbation forces were all sampled at 1000 Hz using a Vicon Mx Giganet, which synchronized the analog and video data.

### Frontal plane force perturbations

**Fig 1:**
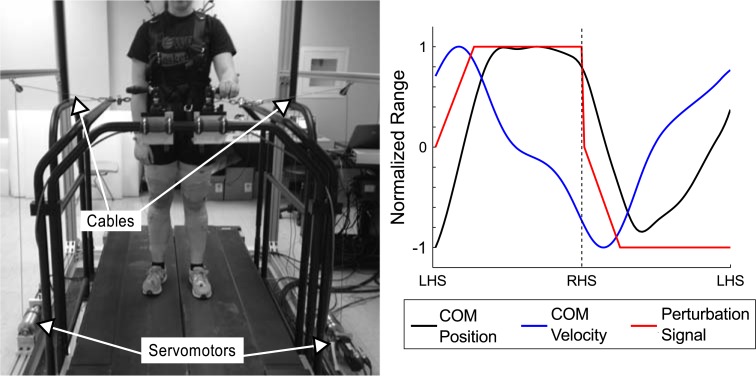
Experimental setup. Participants walked on a split-belt treadmill at their self-selected speed (belts tied at the same speed). Two servomotor systems were used to drive a cable spool, with the cables connected to the waist belt on the fall arrest harness. An example perturbation timing for a subject is displayed along with the COM position and velocity for that cycle. Perturbation timings were based upon time between successive heel strike events, with perturbation magnitude determined by the participant’s body weight.

A cable-driven device (similar to [17]) was constructed to deliver medial-lateral forces to the trunk during treadmill walking. The cable-driven device consisted of a servomotor system (AKM-33H, AKD-0606, Kollmorgen, Radford, VA) turning an aluminum spool with a light stainless-steel cable attached (Fig 1) that can deliver pulls up to 100 N. A cable-driven device was placed on the left and right side of the treadmill to deliver both left and right forces. Each cable ran through a pulley and attached to the belt of the fall arrest harness worn by the individual. The harness and pulley were height adjusted to have the cable connections near the top of the pelvis. This location enabled us to deliver external forces near the approximate vertical location of the participant’s center of mass.

Applied forces were controlled using a custom LabVIEW (National Instruments, Austin, TX) program to time the perturbation forces to the participant’s walking pattern. This program identified the approximate timing of heel strike events in real time using the center of pressure (COP). We used the COP signal to detect large changes in the derivative of whole body medial-lateral sway, which occurs as the participant begins to shift their weight in early stance. Unlike detection methods that rely solely upon vertical ground reaction forces, this approach ensured that step events would be detected even if the participants placed both feet on the same treadmill belt. The times between successive steps were calculated on a step-by-step basis, and a running average of the past ten steps was used to time the motor pulls with the participant’s walking pattern. After heel strike detection, the perturbation force increased from the baseline force (~6 N) to the peak force over a duration equal to one quarter of the average time between the opposite limb heel strike. The peak force remained constant until the opposite limb heel strike was detected, and the force was then returned to the baseline level. The same force profile was used for both stabilizing and destabilizing forces, but the direction of pulls was reversed. For example, after right heel strike when the COM is moving from left to right, a destabilizing force would pull the COM to the right to exaggerate COM movement. In contrast, a stabilizing force would pull the COM to the left after right heel strike to reduce COM movement. This resulted in the timing of the perturbation profile being phased with the medial-lateral COM velocity, while the magnitude of the perturbation was determined by the participant’s body weight.

### Experimental protocol

Walking trials were conducted at the participant’s self-selected treadmill walking speed, which was determined by slowly increasing the belt speed during an initial familiarization trial until the participant verbally indicated a comfortable pace. The initial two walking trials were used to assess baseline walking over a total of 100 gait cycles per leg, first without the cables connected to the participant and then with the cables connected. These two trials enabled the characterization of any changes in the walking pattern related to the bilateral, baseline force (~6 N) necessary to keep the cables taut during walking. Adaptation trials were conducted while the participant walked at their self-selected treadmill walking speed for a total of 312 gait cycles. Each trial was divided into three blocks of 104 gait cycles, with forces applied to the frontal plane during the middle block. This block design enabled characterization of walking changes before, during, and after forces were applied. The use of step-by-step forces allowed for characterization of the time course of any adaptations and/or de-adaptations to the perturbations. Additionally, four catch trials were included in each block that occurred randomly every 25–35 steps. During these catch trials, the forces were either applied (pre and post blocks) or removed (pull block) for a single gait cycle, to further characterize any adaptations. A total of four adaptation conditions were tested: two force magnitudes, 2.5% and 5% of the participant’s body weight (BW), timed to either destabilize or stabilize COM motion. Additionally, the effects of a handrail hold were tested by having the participant either hold onto the instrumented handrail or to hold onto the harness chest straps with both hands. These experimental conditions resulted in a total of eight test trials (2 force magnitude x 2 force type x 2 hand hold) that were presented in a randomized order. A final normal (no applied forces) walking trial of 100 gait cycles with the cables connected was completed at the end of the experiment to evaluate any residual changes in walking performance from the applied forces.

### Data analysis

Video data were initially processed in Vicon Nexus software to label markers and run the lower extremity Plug-In-Gait model. Gait events were automatically detected in Matlab (Mathworks, Natick, MA) using a custom algorithm that combined ground reaction force and kinematic event detection methods described by Zeni et al. [18]. An eight-segment model consisting of the foot, shank, thigh, pelvis, and trunk was used to estimate whole body COM location [19]. Note that trunk motion was estimated using the 3D marker positions, which were then used to calculate the segment COM using the equations described by Winter [19]. Whole body COM was then calculated by summing the separate segment 3D segments. COM sway was used to characterize the magnitude of frontal plane movement of the COM and was calculated as the range of COM movement in the frontal plane over each gait cycle. Temporal (cycle duration and cadence) and spatial (step width) gait parameters were calculated to characterize locomotor adaptations in response to the external COM perturbations.

Locomotor adaptations in COM sway and step width in response to the applied perturbations were quantified utilizing three different analyses. Responses during the catch trials were compared to the to the average response from the corresponding block. Single-cycle perturbation catch trials in the pre and post blocks were compared to the average response across the pull block to assess differences in locomotor adaptions between a single-cycle and prolonged step-by-step perturbations. Catch trials in the perturbation block where the perturbation was removed for a single cycle were compared to the average across the pre and post perturbation block to evaluate retention of the adaptation. Longer term adaptations over the course of the perturbation block were characterized by comparing the average of the first twenty gait cycles with the final twenty gait cycles of the perturbation block. Lastly, we characterized time course of adaptation to the application and removal of perturbations by fitting an exponential curve to the first 20 gait cycles of the perturbation (adaptation) and post perturbation block (de-adaptation). Catch trials were removed, trial data was normalized to the average of the pre-block for a given condition, and then averaged across participants, resulting in a value of one representing the baseline value for a given trial. A Levenberg-Marquardt least squares optimization was used to fit the exponential functions to the data, with the corresponding time constants obtained from these fits used to characterize when the data approached 67% of the steady-state level. All data analysis was completed in Matlab.

Statistical analyses were conducted using SPSS 20.0 (IMB, Armonk, NY). Paired t-tests were used to compare step width and COM sway between the two initial walking conditions, and to assess any differences due to connecting the cables to the participant. The average response was calculated for each testing block (pre, pull, post was obtained by removing catch trials, then taking the mean across the entire block (100 gait cycles). Values from the catch trials were averaged within each block (4 gait trials) for statistical analysis. These average values for step width and COM sway were compared using a repeated measures ANOVA to characterize within-subject changes due to the experimental factors of force type (destabilizing vs. stabilizing; Type), force magnitude (Force), handrail hold (Hold), and perturbation block (Block). A repeated measures ANOVA was also used to compare catch trial responses (step width and COM sway) to the corresponding perturbation block response (i.e. catch trials in the perturbation block were compared to the pre and post blocks since no perturbation was applied for any of these cycles). This analysis method enabled the evaluation of potential interaction effects between the testing conditions, such as the influence of handrail hold during the perturbation block. If the data for a certain experimental factor was not spherical, a Greenhouse-Geisser correction was used for the within-subject effects. Post-hoc analyses were carried out for significant factors using a Bonferroni correction to account for multiple comparisons. Significance was accepted at p<0.05.

## Results

### Device evaluation

**Fig 2:**
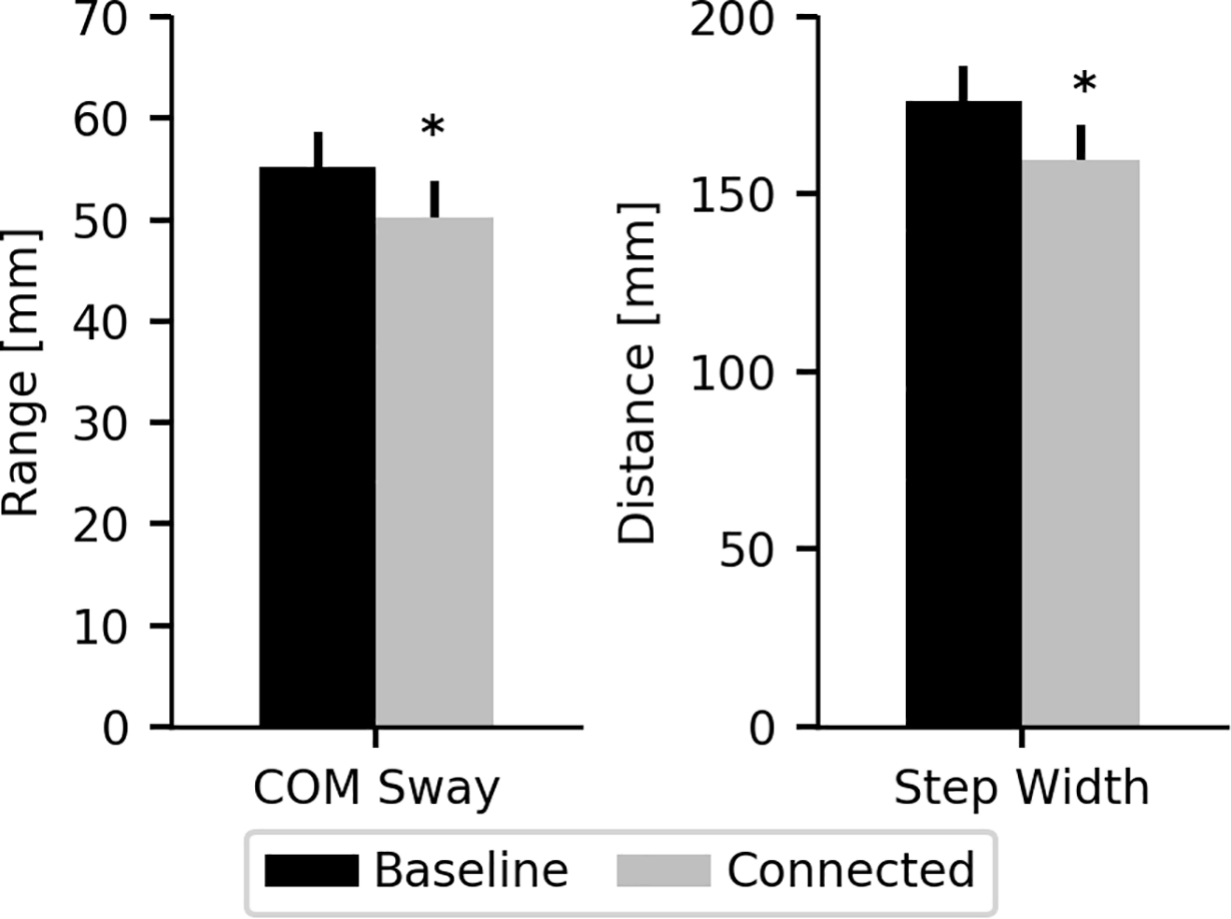
Cable tension alters walking. Cable forces needed to maintain tension within the cables resulted in significant decreases in frontal plane COM movement and step width (* p<0.05, paired t-test).

We evaluated whether connecting the individual to the cable-driven device altered their walking because of the forces necessary to maintain cable tension. Significant decreases in COM sway (p<0.001) and step width (p<0.001) were observed when the cables were connected to the trunk (Fig 2). Although both cables pulled equally with a light (~6 N) force, this tension force altered COM movement and foot placement in the frontal plane. Locomotor changes were assessed within each experimental trial and not with respect to the baseline, un-connected, walking trials.

**Fig 3:**
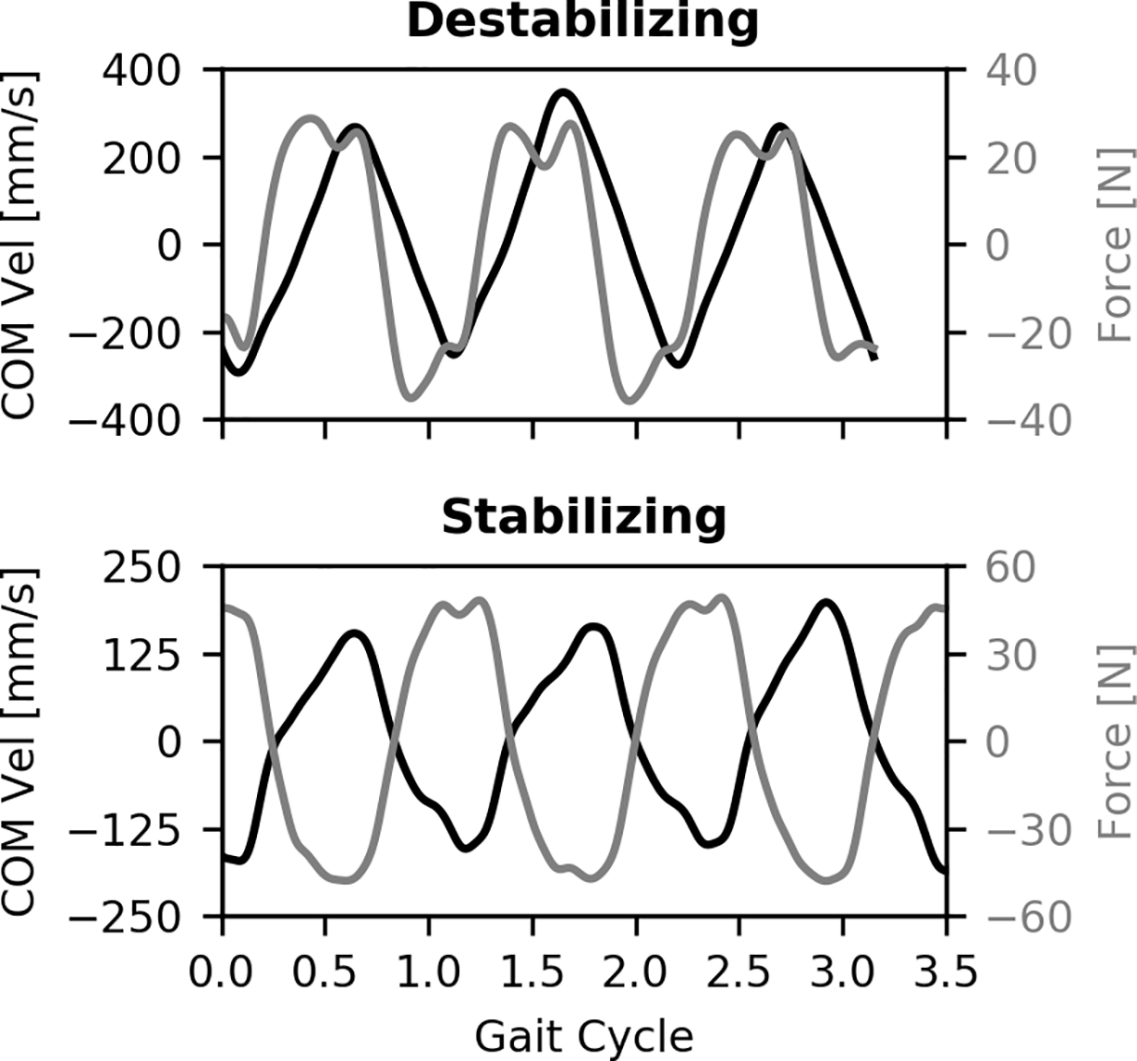
Perturbation force timing. COM velocity (black line) and net applied force (grey line) from three consecutive gait cycles in the perturbation block from a single participant. The applied force was in phase with the COM velocity during the destabilizing perturbations, and 180° out of phase with COM velocity for the stabilizing perturbations.

The custom control program was able to correctly count the number of steps taken with each leg, despite occurrences when the participant simultaneously stepped on both treadmill belts. There was a slight delay between the event identified from the COP and the actual heel strike event from the vertical ground reaction forces (~250 ms), but the observed cycle and step times were similar. Using the observed step times resulted in a force profile that phased with COM velocity in the frontal plane. The destabilizing forces were in phase with COM velocity, while the stabilizing forces were approximately 180° out of phase with COM velocity (Fig 3). The cable-driven device was able to deliver controlled forces on frontal plane COM that synchronized with individual walking patterns.

**Fig 4:**
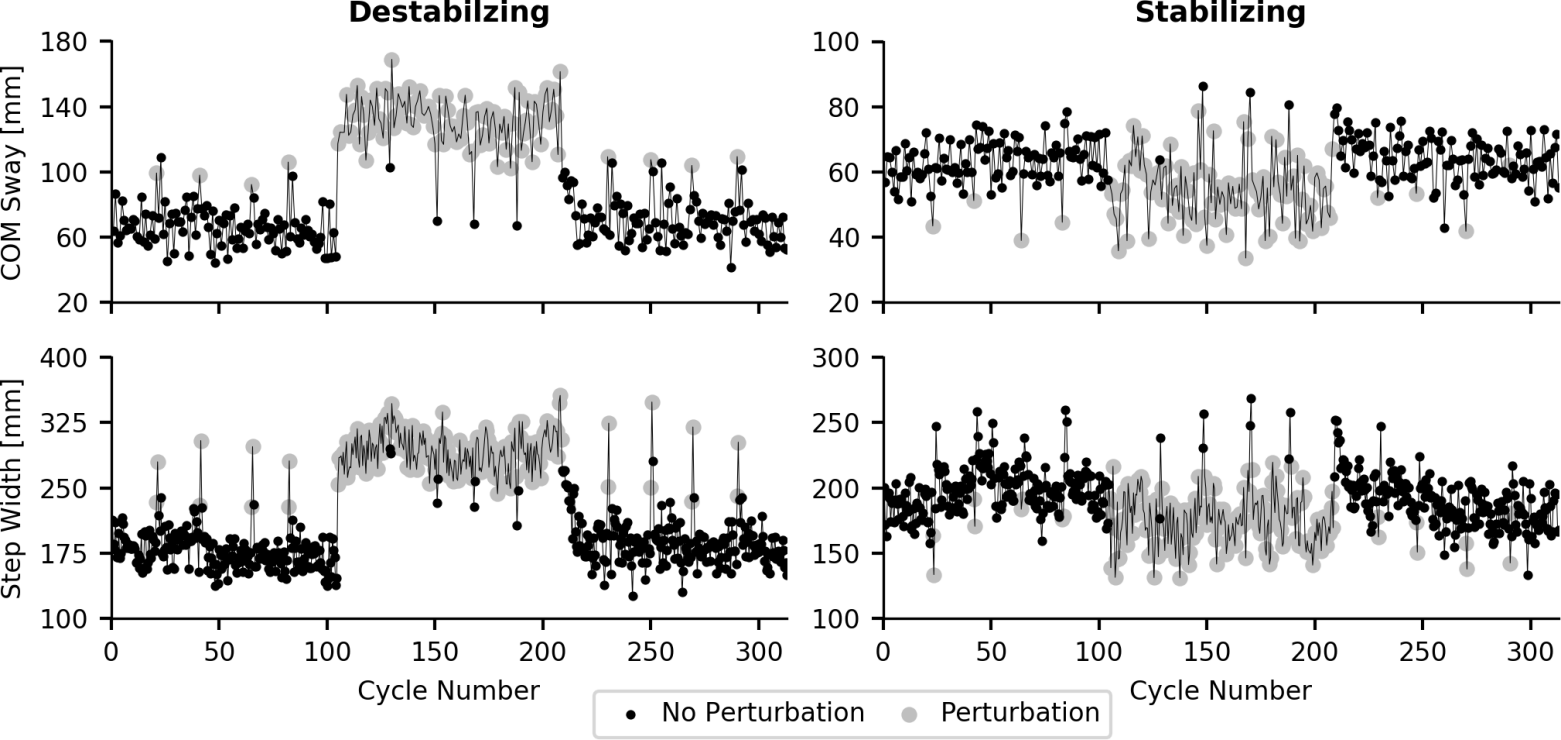
Example response to perturbation from a single participant. COM Sway and step width from a destabilizing perturbation (5%BW no hold) and stabilizing perturbation (2.5%BW no hold) trial. Grey circles indicate perturbation was applied during the gait cycle. Both COM sway and step width increased in response to the accentuating perturbation condition and decreased when perturbations resisting COM movement were applied.

Example step widths and COM sway for two representative participants are presented in Fig 4. The destabilizing and stabilizing forces had opposite effects on trunk movement. This difference resulted in a significant main effect of force type for COM sway (p<0.001), step width (p<0.001), and cycle duration (p<0.001). To reduce the number of significant interaction effects, separate repeated measures ANOVAs were carried out for each force type. The results for each force type are presented separately below.

### Response to destabilizing trunk forces

**Fig 5:**
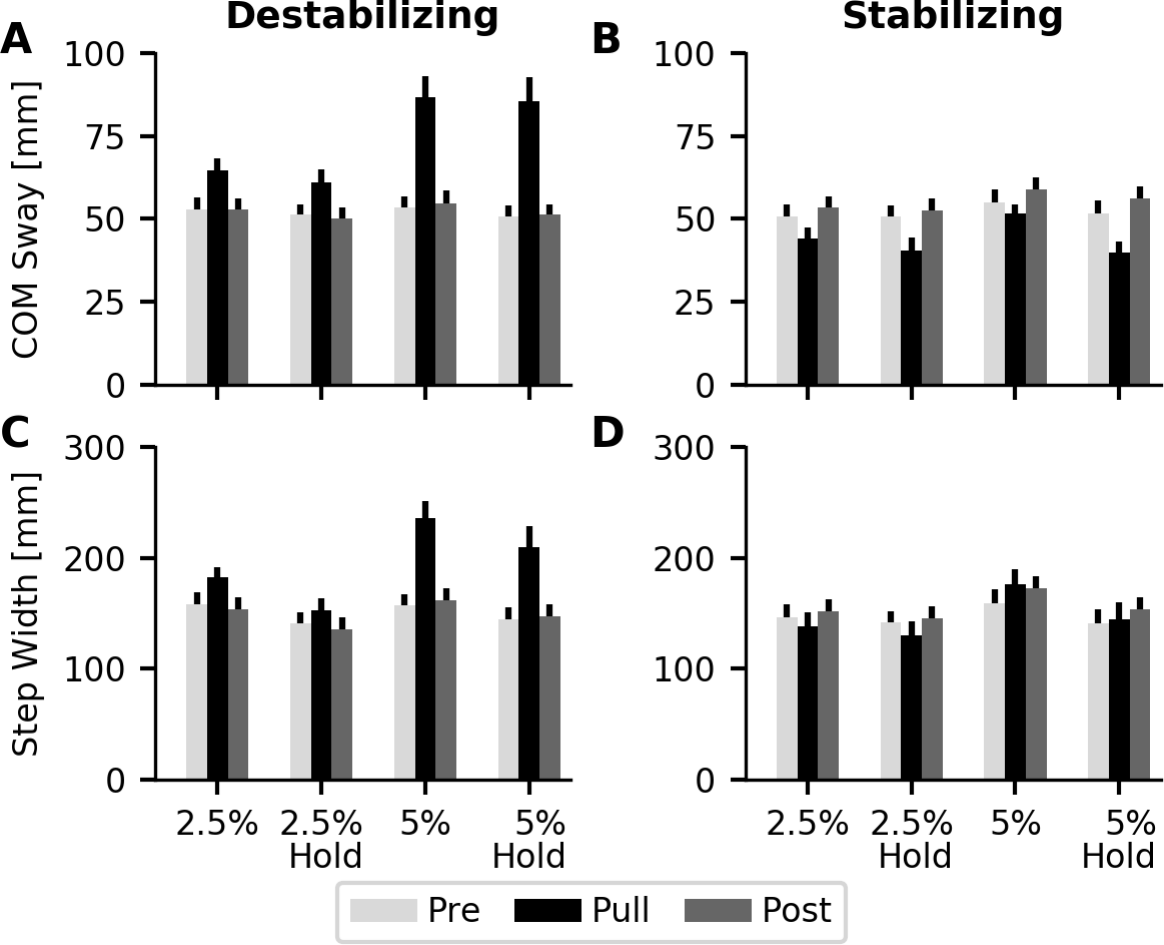
Average COM sway and step width response to perturbations. The destabilizing perturbations increased COM sway (A) and step width (C), with larger changes observed at higher perturbation forces. Conversely, the stabilizing perturbations reduced COM sway (B), with smaller reductions observed in step width (D). Handrail hold had a general effect of reducing step width across the entire trial, but a significant effect was only observed for the stabilizing forces.

Comparison of the group average COM sway within each testing block for the destabilizing condition revealed significant main effects of force magnitude (p = 0.002) and testing block (p<0.001), as well as a significant interaction effect between these two main effects (Force*Block, p <0.001). COM sway was significantly higher in the applied force block compared to the pre and post (p<0.001) blocks, while no average differences in COM sway were observed between the pre and post blocks. Increasing the magnitude of the force from 2% BW to 5% BW increased the amount of COM sway in the force block (Fig 5A). The interaction between force magnitude and testing block is likely due to the increase in sway during the force block, since no differences were found between the pre and post perturbation blocks for the two force magnitudes. There was no significant effect of handrail hold observed for the destabilizing force (p = 0.074).

The observed increased COM sway was accompanied by increases in step width when destabilizing forces were applied. Main effects of force magnitude (p = 0.015), handrail hold (p = 0.001), and testing block (p<0.001), and an interaction between force magnitude and testing block (Force*Block, p = 0.003) were observed. Handrail hold had a general effect of reducing step width across the three testing blocks, with no significant interactions observed for force magnitude (Hold*Force, p = 0.574) or testing block (Hold*Block, p = 0.067). The destabilizing forces resulted in larger step widths during the applied force block compared to the pre (p = 0.001) and post blocks (p = 0.001) (Fig 5C). As force magnitude increased, step width also increased, but only during the applied force block. No significant differences were observed between the pre and post blocks for step width.

In addition to the changes in frontal plane gait parameters, cycle duration was also decreased when destabilizing forces were applied. The application of destabilizing forces resulted in decreased gait cycle duration (i.e. increased cadence) during the applied force block compared to the pre (p<0.001) and post (p<0.001) blocks. As the force magnitude increased, the cycle duration further decreased, but only when the force was applied. Holding onto the handrail had the general effect of slightly increasing gait cycle duration (decreasing cadence) across all testing blocks (p = 0.001).

### Response to stabilizing forces

The stabilizing forces acted to reduce COM sway when applied during walking (Fig 5B). Significant main effects of force magnitude (p = 0.013), handrail hold (p = 0.003), and testing block (p <0.001) were observed. Additionally, interaction effects were observed between handle hold and block (Hold*Block, p < 0.001), and handle hold, force magnitude, and block (Force*Hold*Block, p = 0.005). When the force was applied during walking, COM sway was reduced compared to the pre (p = 0.005) and post force blocks (p = 0.001). Removal of the stabilizing forces resulted in larger amounts of COM sway compared to the pre-perturbation block (p = 0.004). Holding onto the handrail caused further reductions in COM sway, but this effect only occurred during the applied force blocks.

In contrast to the destabilizing forces, changes in COM sway were not coupled with step width changes for the stabilizing force field (Fig 5D). An interaction between force magnitude and testing block (Force*Block, p = 0.031) was likely due to an observed trend towards reduced step width at the 2.5% BW force magnitude, but not 5% BW. Similar to the destabilizing forces, handrail hold had the general effect of reducing step width across the entire trial (p<0.001).

The stabilizing forces acted to increase gait cycle duration (Block, p = 0.006), with the perturbation (p = 0.011) and post (p = 0.001) testing blocks having a longer cycle duration (lower cadence) compared to the pre-perturbation block. Handrail hold further increased gait cycle duration when perturbations were applied (p = 0.001).

### Locomotor adaptations

We found no significant differences in the average COM sway or step width between the pre and post perturbation blocks for either perturbation type. This return of step width and COM sway to baseline levels once the perturbation was removed suggest there was no significant shift in locomotor control strategy during unperturbed treadmill walking.

**Fig 6:**
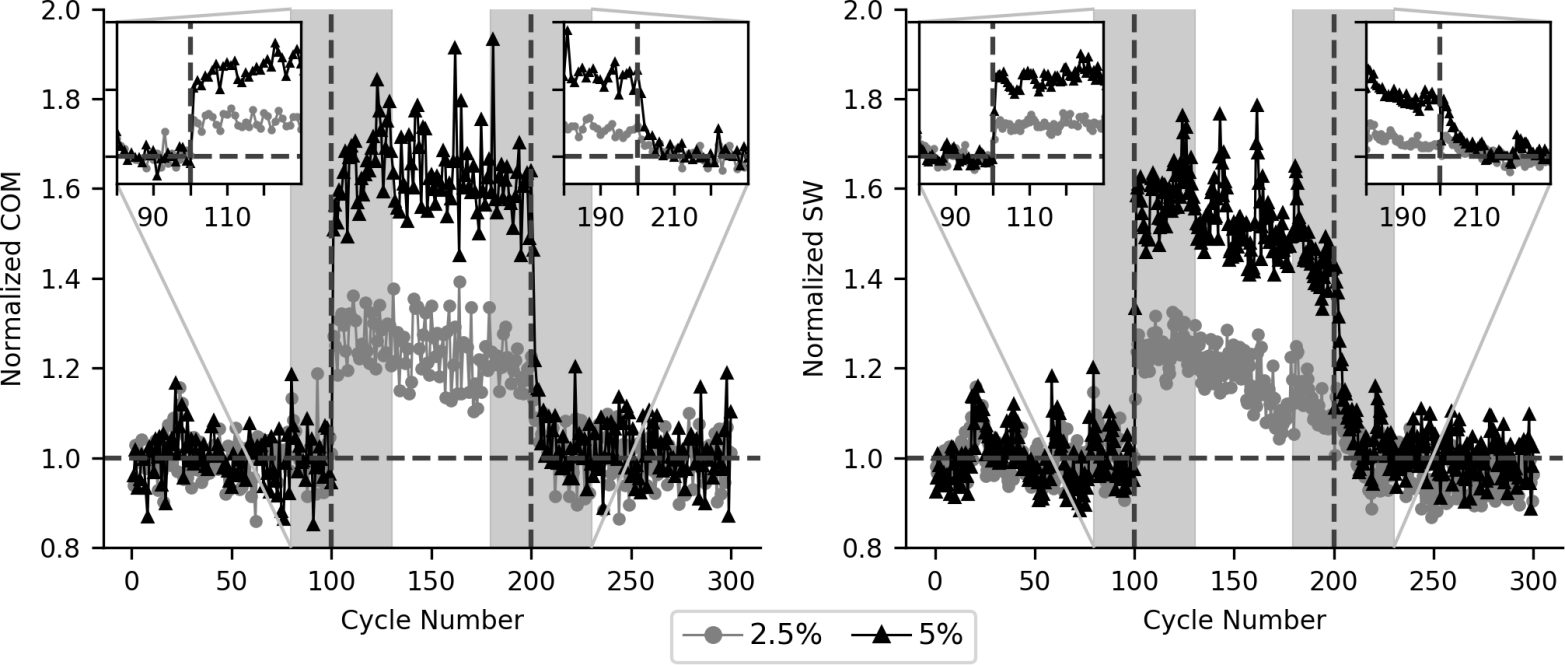
Time course of locomotor changes to destabilizing forces. Ensemble averaged group response to destabilizing force field without the handrail hold. Catch trials were removed and values were normalized to each participant’s average response in pre perturbation block prior to ensemble averaging. Perturbations were applied during cycles 101 to 200. Inset graphs display response at transition points of perturbation (shaded regions). COM sway and step width quickly increased when perturbations were applied, and quickly returned to baseline levels when perturbations were removed.

Group average COM sway and step width over the entire trial, and a subset of the steps at the block transition points for the destabilizing, no-hold perturbation type are shown in Fig 6. Examination of the temporal components of this adaptation suggests both a transient component at the application and removal of the perturbations (Fig 6 inserts), and a steady state component over the course of the perturbation block. The steady state component was captured by comparing the average of the first and last 20 cycles of the perturbation block. No significant differences were observed in COM sway between the start and end of the perturbation block for either destabilizing forces (Block, p = 0.558) or stabilizing forces (Block, p = 0.327). However, step width significantly decreased over the course of the perturbation block for destabilizing forces (Block, p = 0.013), but no significant change was observed for the stabilizing forces (Block, p = 0.389). Visual inspection of the transient response suggests both step width and COM sway rapidly increased in response to the destabilizing forces, but took longer to return to baseline levels when the destabilizing forces were removed (Fig 6 insets). Quantification of the transient component of the locomotor adaptations was impacted by the inclusion of a catch trial within the first 25–30 steps of each block, as well as step by step variability in the measured responses. Given these limitations we limited our analysis to the destabilizing perturbation at 5% BW with no handrail hold, which produced the largest change in COM sway and step width. Both COM sway and step width rapidly adapted to the application of the destabilizing perturbation, with a time constant of 0.83 for COM sway and 0.44 for step width. These values indicate that when the destabilizing forces were applied COM sway and step width were quickly adjusted to almost the steady state value in the initial one to two gait cycles. Removal of the perturbation forces resulted in a time constant of 2.07 for COM sway and 4.81 for step width, suggesting a slightly longer time to reach baseline values.

**Fig 7:**
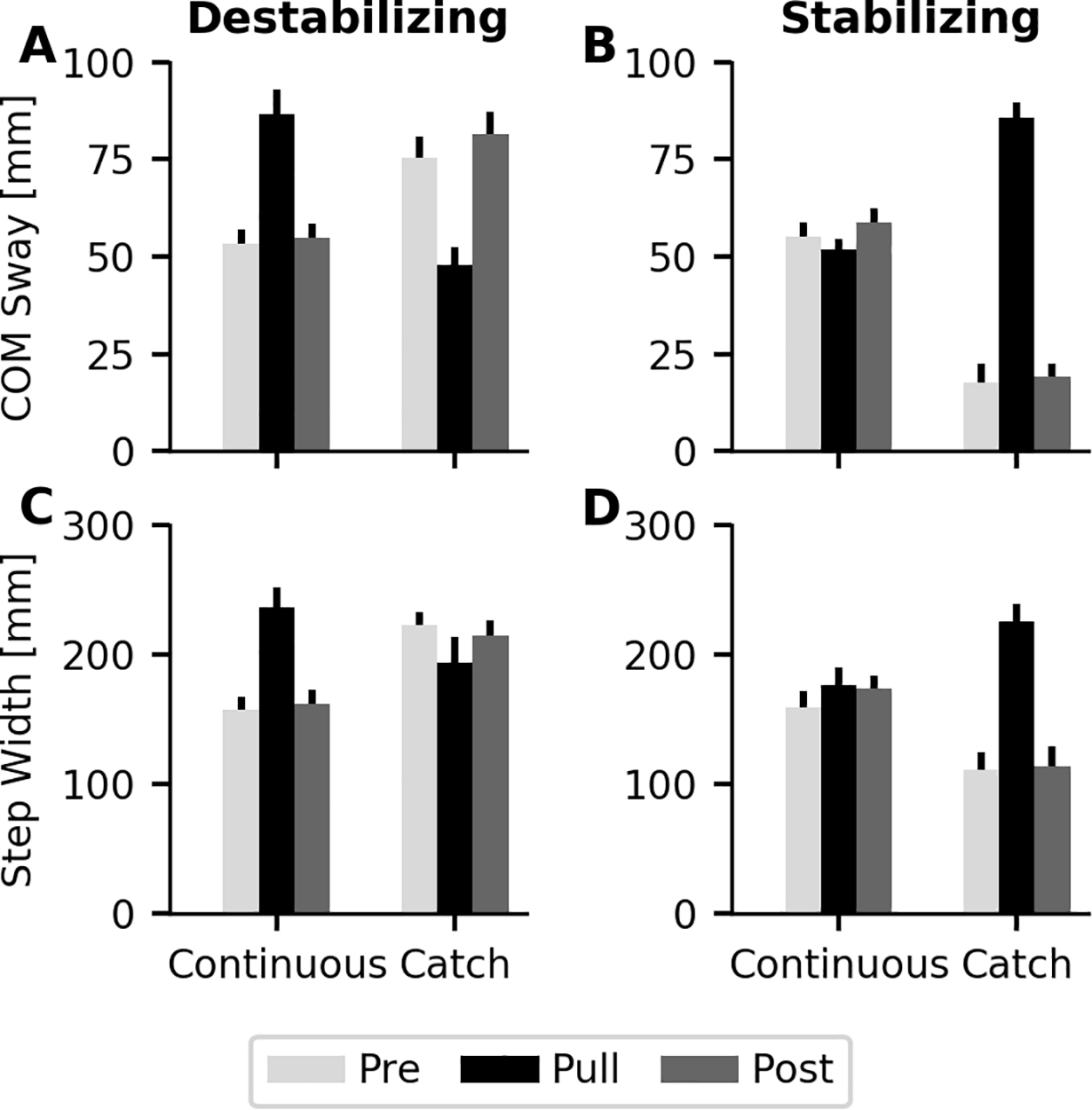
Catch trials for 5% BW and no handrail hold condition. Average catch trial response from the destabilizing (A and C) and stabilizing (B and D) perturbation trials. Catch trials during pre and post blocks applied forces for a single cycle, while catch trials in the perturbation block removed applied forces for a single cycle. Destabilizing forces demonstrated an additive effect with prolonged step-by-step perturbations producing larger changes compared to single cycle perturbations. Single cycle stabilizing perturbations produced larger changes compared to prolonged step-by-step perturbations likely due to the perturbation of COM movement opposite to the direction of movement. Removal of forces in the perturbation block demonstrates retained changes in both COM sway and step width for both perturbation types, indicating participants did adapt to the applied forces.

Comparison of single cycle catch trial responses during the pre and post perturbation blocks with responses from the perturbation block provided a measure of differences in adaption to single-cycle and prolonged step-by-step perturbations. Increasing the perturbation force increased the magnitude of changes in both COM sway and step width for destabilizing and stabilizing forces (all p’s<0.05 for Force and Force*Block). Single cycle applied destabilizing forces resulted in smaller increases in both COM sway and step width compared the perturbation block, suggesting an additive effect of the perturbations (Fig 7A and 7C). In contrast, single cycle applied stabilizing forces produced larger decreases in both COM sway and step width compared to prolonged, step-by-step perturbations (Fig 7B and 7D). No significant differences were observed between the pre and post perturbation block catch trials for either force, suggesting similar response to single cycle applied forces before and after exposure to the force field.

Catch trials removing applied forces during the perturbation block, provided a measure of retained locomotor adaptations to applied forces. COM sway during the catch trials was significantly lower than baseline when destabilizing forces were removed (Fig 7A; Block, p = 0.033), suggesting an adaptation in COM control. Additionally, removal of 5%BW destabilizing forces in the perturbation catch trials resulted in greater step widths compared to baseline (Fig 7B; Force*Block, p<0.001). Contrastingly, removal of stabilizing forces produced greater amounts of both COM sway and step width (Fig 7B and 7D), with the largest change observed for the 5%BW force level (Force*Block, p<0.001).

## Discussion

Results from this experiment demonstrate changes in gait parameters with frontal plane forces applied to the trunk. Forces intended to resist COM movement in the frontal plane (stabilizing force) decreased COM sway, while forces designed to accentuate COM movement (destabilizing forces) increased COM sway for all participants. Participants were able to adapt to these external perturbations, maintaining dynamic balance by increasing step width in response to destabilizing forces. Interestingly, step width did not decrease significantly with applied stabilizing forces. In addition, cadence changed with the applied forces, increasing for destabilizing forces and decreasing for stabilizing forces. These locomotor changes indicate that while changes in lateral foot placement (step width) is the primary source of adaptation to the applied forces, individuals, in part, utilized a COM control strategy (cadence). Retained differences in both COM sway and step width during catch trials where the applied forces were removed, demonstrated that individuals adapted to the external forces. Additionally, the timing of these adjustments indicated that the dynamic balance control strategy quickly reacts to conditions challenging balance, while taking a more conservative approach to conditions reducing balance demands.

This study provides a unique insight into how individuals adapt to step to step destabilizing forces in the frontal plane. Medial-lateral forces have been previously applied during walking, demonstrating that individuals take a wider step to account for the increased lateral trunk movement [15]. However, these perturbations were only over a single step and do not provide insight into the time course of the adaptations. Similar to previous studies, we observed that participants primarily modified lateral foot placement to account for changes in COM movement. Prolonged step-by-step destabilizing forces resulted in larger step width increases compared to those observed during the single cycle catch trials. Most of the foot placement adaptation was achieved over one or two gait cycles, while de-adaptation to the destabilizing perturbations took approximately five to ten cycles to return to baseline (Fig 6). Reisman et al. [9, 20] observed that intralimb parameters (stance time and stride length) quickly adapted to split-belt speed perturbations and demonstrated post-adaption aftereffects, while interlimb parameters (double support time) slowly adapted with no aftereffects. They hypothesized that the slower rates of adaptation in the interlimb parameters are used to restore a symmetric walking pattern, after the intralimb parameters change to adjust to the speed differences. Overall, in our study we observed much faster rates of adaptation and de-adaptation than the rates observed for the interlimb parameters during split-belt walking [9] and other walking adaptation paradigms [21–24]. These fast adaptation rates are likely due to the need to quickly adjust the base of support to prevent a fall, supporting the theory that the central nervous systems focuses more on controlling frontal plane balance [3]. However, we did observe relatively slower de-adaptation rates for all destabilizing conditions, as well as a significant decrease in step width over the course of the perturbation block at the 2% force level. These observations suggest that dynamic balance control takes a more conservative approach to reducing the base of support when balance demands are reduced.

Analysis of the catch trials provided further insight into the locomotor adjustments made in response to the balance perturbations. Similar responses were observed when destabilizing perturbations were applied over one cycle compared to prolonged step-by-step perturbations. Application of the stabilizing forces for a single cycle produced larger decreases in COM sway and step width compared to prolonged step-by-step forces. This difference is likely due to participants taking a quick step to maintain balance in response to the perturbation pulling the COM back towards the swing leg. Removal of forces for one cycle resulted in lower levels of COM sway in the destabilizing condition and higher levels of COM sway in the stabilizing condition, when compared to average levels in the pre and post perturbation blocks. These changes in COM sway indicate that participants are making other locomotor adjustments to control COM movement in response to the external trunk perturbations. The observed changes in gait cycle duration during the perturbation blocks at least partially explain the differences in COM sway when perturbations were removed, since the gait cycle duration influences the extent of COM movement [25]. Differences in COM sway when both the destabilizing and stabilizing forces were removed, indicate that although step width control was the primary mechanism for dynamic balance control, participants also made spatial and temporal adjustments to help control COM movement. Specifically, when stabilizing forces were applied, cadence decreased and when destabilizing forces were applied cadence increased. These findings are in agreement with work done by Hak et al (2013), that demonstrated that medial-lateral margins of stability increase as individuals walked at a faster cadence [26].

Additionally, step width responses during the catch trials removing 5% stabilizing forces were consistent with observations from the applied forces. Step width quickly increased in response to the perceived increase in COM sway when the stabilizing forces were removed. Removal of the destabilizing forces resulted in the persistence of larger step widths than baseline, despite the decreased COM movement. These catch trial responses demonstrate that base of support adjustments are rapidly made in response to challenging balance conditions, and are slower to return to baseline levels when the challenge is removed.

The effect of handrail hold on the magnitude of the locomotor adaptations provides important insight for future studies. Treadmill walking studies often involve the use of the handrails to ensure participant safety, especially for the elderly or individuals with a neurologic disorder. Handrail hold has been shown to reduce step length and width variability during treadmill walking [27]. Additionally, a light touch force when using a cane is sufficient to stabilize movement of the pelvis in stroke survivors [28]. Therefore, it is possible that holding onto the handrail could significantly alter how individuals adjust to balance perturbations. We observed a trend towards the handrail hold impacting step width responses to the destabilizing (Hold*Block, p = 0.067) and stabilizing (Hold*Block, p = 0.08) forces. The handrail hold augmented locomotor changes in step width and cycle time when stabilizing forces were applied. However, handrail hold may have a more significant effect for individuals with neurological disorders, since the handrail could be used to provide postural support or assist with controlling COM movement.

Adaptations to step width in the current study were likely driven by a change in the balance between stability and the energetics of lateral stabilization. Participants produced narrower step width in the stabilizing force field and wider step width in the destabilizing force field. Step width is a prime determining factor for stabilization during gait, with higher metabolic costs generally associated with wider step widths [29]. The addition of lateral springs that stabilize the trunk decreases step width, similar to stabilizing force field in the current study, and reduces energy expenditure during walking [30–32]. With measurements of applied force and COM displacement, it would have interesting to identify the timecourse of changes in energy exchange between the apparatus and the participant during the adaptation period. Unfortunately, attempts to calculate energy exchange were complicated by unknown mechanical properties of the machine-person interface and artifacts in the force signals. In future studies a redesigned interface might make it possible to calculate the timecourse of adaptations to the forcefield based on the energy exchange.

In conclusion, these results validate the use of the cable-driven system to create novel balance environments to study dynamic balance control during walking. This study has demonstrated that young healthy participants were able to make the necessary modifications to step width to maintain dynamic balance during treadmill walking in the presence of prolonged step-by-step frontal plane stabilizing and destabilizing forces. Application of destabilizing forces produced more robust balance adjustments, which were not strongly influenced by holding onto the handrail. Differences in the rates of adaptation and de-adaptation for the destabilizing perturbations suggest that dynamic balance control prioritizes adjustments to prevent falls but is more time conservative with adjustments when the balance demands are lessened. Additionally, this device may be useful to examine changes in dynamic balance control strategy for individuals with neurological disorders, such as stroke.

## Supporting information

S1 Table **[COM_block]** Center of mass (COM) sway data for each subject are shown. The baseline data were collected during stepping on the treadmill prior to connecting the cables for the force field perturbations and then after connecting the cables with low tension in the cables (6N). COM sway is shown for stabilizing and destabilizing forces at two levels, 2.5% and 5% body weight (BW) when participants were holding (Hold) or not holding (No Hold) the handle at the front of the treadmill. Data are shown for trials before (Pre), during (Pull) and after (Post) the force field block. **[SW_block]** Step width (SW) data for each subject are shown. The baseline data were collected during stepping on the treadmill prior to connecting the cables for the force field perturbations and then after connecting the cables with low tension in the cables (6N). COM sway is shown for stabilizing and destabilizing forces at two levels, 2.5% and 5% body weight (BW) when participants were holding (Hold) or not holding (No Hold) the handle at the front of the treadmill. Data are shown for trials before (Pre), during (Pull) and after (Post) the force field block. **[COM_catch]** Center of mass (COM) sway data for catch trials for each subject are shown. COM sway is shown for catch trials in stabilizing and destabilizing forces at two levels, 2.5% and 5% body weight (BW) when participants were holding (Hold) or not holding (No Hold) the handle at the front of the treadmill. Data are shown for catch trials before (Pre; forces applied), during (Pull; no forces) and after (Post; forces applied) the force field block. **[SW_catch]** Step width (SW) data for catch trials for each subject are shown. SW is shown for catch trials in stabilizing and destabilizing forces at two levels, 2.5% and 5% body weight (BW) when participants were holding (Hold) or not holding (No Hold) a handle at the front of the treadmill. Data are shown for catch trials before (Pre; forces applied), during (Pull; no forces) and after (Post; forces applied) the force field block.
(XLSX)
